# Towards a global scale for functional ability: what gets measured and gets done—but are we measuring the right thing?

**DOI:** 10.1093/ageing/afaf323

**Published:** 2025-11-06

**Authors:** Marisa Nishio, Yuri Ito, Naoki Kondo, Jotheeswaran Amuthavalli Thiyagarajan

**Affiliations:** Department of Medical Statistics, Osaka Medical and Pharmaceutical University, Osaka, Japan; Department of Medical Statistics, Osaka Medical and Pharmaceutical University, Osaka, Japan; Department of Social Epidemiology, Kyoto University, Kyoto, Japan; Department of Sexual, Reproductive, Maternal, Newborn, Child, Adolescent Health and Ageing (LHR), World Health Organization, Geneva, Switzerland

**Keywords:** healthy ageing, measurement, functional ability, older people

## Abstract

Functional ability—‘the health-related attributes that enable people to be and do what they have reason to value’—is the core outcome indicator of the UN Decade of Healthy Ageing (2021–30). Despite its centrality, there is still no globally standardised tool to measure functional ability across five domains defined by WHO: meeting basic needs; learning, growing, and making decisions; mobility; building and maintaining relationships; and contributing to society. Current approaches remain fragmented, limiting efforts to monitor progress and support equitable policy action. This commentary outlines three strategic priorities for addressing this gap. First, a globally validated functional ability scale should be developed, grounded in the WHO’s five-domain framework. This effort must begin with clarifying the concept of functional ability—moving beyond narrow biomedical definitions—to ensure conceptual consistency. Early efforts in China and Japan demonstrate the feasibility of operationalising domain-level constructs, though challenges remain in empirical alignment. Second, this scale must be embedded in WHO global ageing population surveysto ensure cross-country comparability and data coverage in high-low- and middle-income contexts. Third, the field must move towards personalised functional ability profiles, reflecting older adults’ own values and goals. Innovations in AI and machine learning may support this transition. With only five years remaining in the Decade, finalising and implementing a standardised, person-centred measurement approach is urgent. Doing so would not only advance international monitoring but also help realise the vision of enabling older people everywhere to achieve healthy ageing.

## Key Points

- Functional ability is the core outcome of the UN Decade of Healthy Ageing, yet no global standard exists.
- A standardised scale based on the WHO’s five domains should be developed and integrated into global ageing surveys.
- Person-centred measurement, including personalised profiles, is needed to reflect what people value in later life.
- A global measurement framework would enable equitable policy action and monitoring of progress in healthy ageing.

## Midway through the decade of healthy ageing

We have reached the midway point of the United Nations Decade of Healthy Ageing (2021–30), a global initiative aiming to ensure older people can live long and healthy lives [1]. At the heart of this effort is functional ability—defined by the World Health Organisation (WHO) as the combination of ‘health-related attributes that enable people to be and do what they have reason to value’ [2], and recognised as one of the core outcome indicators for monitoring progress throughout the Decade [3]. Healthy ageing centres on maintaining capabilities across five domains: (i) meeting basic needs, (ii) learning, growing, and making decisions, (iii) mobility, (iv) building and maintaining relationships, and (v) contributing [2]. These reflect a person-centred view of ageing, focusing on the ability to live a meaningful life. Yet, a critical gap remains: there is still no globally standardised tool to measure functional ability in all its domains. Current approaches are fragmented and inconsistent across countries, undermining our efforts to track progress and capture person-centred aspirations.

This commentary outlines the measurement problem and proposes three steps: (i) develop a global functional ability scale aligned with WHO’s five-domain model, (ii) integrate this scale into global ageing studies, and (iii) innovate towards personalised functional ability profiles. Population-level functional ability profiles—disaggregated by region or sociodemographic group—can support targeted interventions, resource allocation, and equity-oriented planning. They can help governments identify where support is most needed and how diverse groups are progressing across the five domains of functional ability.

## Do concepts matter? Why definitions shape measurement?

Concepts shape measurement, and measurement shapes action. When functional ability is vaguely or inconsistently defined, tools vary—not only in what they measure but in their underlying assumptions about ageing. A biomedical definition may focus on physical decline [4], whereas WHO’s broader model includes social and cognitive dimensions aligned with the capability approach [1, 2, 5]. Clarity is essential for accountability. Without a shared understanding of functional ability, stakeholders may use inconsistent indicators and report selective progress. If the concept is too vague, it can be diluted or diverted from its original intent; if it is too narrow, it may exclude the core aspect, including what older people value. Aligning definitions with lived experience allows functional ability measures to reflect societal commitments to dignity in ageing. Conceptual discipline is the first step towards building robust, person-centred metrics.

## Complexity and vagueness: the hidden barriers to measurement

The WHO’s five domains of functional ability represent subjective, context-dependent capabilities. For example, ‘contributing’ or ‘making decisions’ vary across cultures and individuals. Indicators that capture these constructs in one setting may not work in another. Zhao *et al*. operationalised functional ability in China, validating five domains through factor analysis but noting challenges in consistently measuring constructs like ‘contributing’ [6]. Similarly, Nishio *et al*. found that while domain-specific constructs were empirically distinguishable in Japan, some—such as ‘learning, growing, and making decisions’, and ‘contributing’, as well as ‘meeting basic needs’ and ‘mobility’—tended to cluster together [7]. The term ‘functional ability’ is used inconsistently across academic, policy, and clinical settings. In OECD contexts, it is often equated with dependency or IADL limitations [4], whereas the WHO adopts a broader capability-based definition [1, 2, 5]. These variations reflect not only definitional inconsistency but also the empirical reality that WHO’s theoretical domains may not always map neatly onto observed data. Bridging this gap will require both conceptual clarity and iterative empirical validation to support the development of standardised yet context-sensitive indicators.

## Towards a global functional ability scale

The first strategic step is an internationally validated scale grounded in WHO’s five-domain model. This would provide a common yardstick for healthy ageing. Initial work in individual countries suggests this is feasible [6, 7]. A global effort is now needed to refine and harmonise a core set of items per domain. WHO’s Technical Advisory Group on Measurement of Healthy Ageing (TAG4MHA) and a recent systematic review have identified candidate questions from international surveys [3, 8]. Yet no single scale has been finalised. As Michel *et al*. argue, developing a global functional ability metric informed by the capability approach is essential for tracking well-being in older age [9]. A standardised yet adaptable scale would allow countries to measure healthy ageing using a common lens and move beyond disparate clinical indicators towards what matters most: people’s ability to live well.

## Embedding the scale in global ageing surveys

Developing a scale is only the beginning. The next step is embedding it in global longitudinal surveys such as the Health and Retirement Study [10] and its sister surveys [11, 12]. These surveys already cover millions of older adults across 40+ countries and offer a ready-made infrastructure. Incorporating a standard functional ability module would support global comparisons and trend analyses. It is also efficient, leveraging existing sampling frameworks. Efforts should be made to extend such surveys to low- and middle-income countries and ensure no older population is excluded from data collection. WHO’s new Global Ageing Population Survey (GAPS) can help fill these gaps, particularly where ageing-specific surveys do not yet exist. Its modular structure offers an opportunity to embed the functional ability scale in underrepresented contexts. WHO’s earlier use of three ADL items as a proxy for basic needs showed that comparability is possible when indicators are aligned [1]. Now, this principle must extend to all domains of functional ability. Integrated global data on functional ability, to be reflected in the 2026 and 2029 UN Decade impact evaluation, will help identify countries and domains requiring further policy attention.

## Looking forward: personalising functional ability measurement

Looking ahead, we must complement population-level measures with personalised functional ability profiles. Healthy ageing is highly heterogeneous: some 80-year-olds are as fit as those decades younger; others require support. Assessments should reflect personal goals and preferences as well as domain scores. The WHO’s Healthy Ageing concept distinguishes between achieved function and the capability to achieve. Evaluating what older people value—playing with grandchildren, community involvement, or lifelong learning—should become part of how we assess functional ability. Emerging technologies can support this. Smartphones, wearables, and digital platforms can track real-time data on mobility, social interaction, and cognition. Machine learning may help identify patterns—e.g. high physical function but low social participation—prompting tailored interventions. Person-reported outcome tools could also enable individuals to set and evaluate their own ageing goals. Although full personalisation may be achieved beyond 2030, laying the groundwork now is critical. Pilot programmes and research on AI-based prediction models can inform future refinement of the global scale. In this way, person-centred care becomes embedded in the measurement system itself.

## Seizing the second half of the decade

With only five years left, the opportunity to build a global functional ability measurement infrastructure is narrowing. Functional ability must not remain a noble but unmeasured concept. The steps outlined—finalising a global scale, embedding it in international surveys, and exploring personalised approaches—are mutually reinforcing. As WHO experts emphasise, ‘measures, data collection, analysis and reporting are urgently needed to support global, regional and national monitoring’ [13]. The conceptual, technical, and institutional foundations are in place. What is needed now is the political and scientific will to act. By doing so, we can ensure the Decade’s legacy is not merely an extended lifespan, but the realisation of what matters most to older people: the ability to live well, with dignity and autonomy. The measurement of functional ability will be central to that legacy.
